# Social support mediates the relationship between depression and subjective well-being in elderly patients with chronic diseases: Evidence from a survey in Rural Western China

**DOI:** 10.1371/journal.pone.0325029

**Published:** 2025-06-02

**Authors:** Zhonglian Li, Suxia Qin, Yafen Zhu, Quanxiang Zhou, Aijing Yi, Caiyun Mo, Jun Gao, Juhai Chen, Tianhui Wang, Zhanhui Feng, Xiangang Mo

**Affiliations:** 1 School of Clinical Medicine, Guizhou Medical University, Guiyang, China; 2 Department of Medicine, Qiannan Medical College for Nationalities, Duyun, China; 3 Department of Internal Ward, Guiyang Public Health Service Center, Guiyang, China; 4 Department of Neurology, Guizhou Provincial People’s Hospital, Guiyang, China; 5 Department of Comprehensive Ward, The Affiliated Hospital of Guizhou Medical University, Guiyang, China; UofM: The University of Memphis, UNITED STATES OF AMERICA

## Abstract

**Background:**

The aging population has led to a marked increase in the prevalence of chronic diseases among the elderly, significantly impacting their physical and mental health, as well as their overall quality of life. In rural regions of Western China, these challenges are exacerbated by limited access to medical insurance, low living standards, and inadequate mental health services. Consequently, the physical and mental well-being of elderly individuals with chronic conditions in these areas warrants focused attention. This study aims to investigate the interrelationships between depression, social support, and subjective well-being, with particular emphasis on the mediating role of social support.

**Methods:**

This cross-sectional study involved a survey of 2,156 elderly individuals aged 60 and above, all living with chronic diseases in the rural areas of Qiannan, Guizhou, China. Pearson correlation and hierarchical linear regression analyses were employed to explore the relationships between the variables. A structural equation model was then constructed using Amos 23.0, based on the identified correlations between depression, social support, and subjective well-being. The bootstrap estimation method was applied to assess the mediating effect of social support in the depression-subjective well-being relationship.

**Results:**

The analysis revealed a significant negative correlation between depression and subjective well-being, while social support showed a strong positive association with subjective well-being. Mediation analysis confirmed that social support significantly mediates the relationship between depression and subjective well-being, accounting for 10.23% of the total effect. Notably, the influence of subjective support on depression and subjective well-being was found to be more pronounced than that of objective support or social support utilization.

**Conclusions:**

The findings highlight the necessity of strengthening the social support system for elderly individuals with chronic diseases in rural Western China, particularly by enhancing psychological and emotional support. This approach is crucial for mitigating depressive symptoms and improving subjective well-being in this population.

## Introduction

With the acceleration of the global aging trend, the pace of population aging in China is rapidly increasing [1]. Recent data reveals that the population aged 60 and above in China has reached 297 million, representing 21.1% of the total population [2]. Notably, the rural elderly population constitutes 23.81% of the total rural population, reflecting a more significant aging issue in rural areas [3]. As the population ages, the prevalence of chronic diseases among the elderly has steadily risen. According to the WHO, individuals aged 60 and over account for nearly one-fourth of the global chronic disease burden [4]. In China, over 180 million seniors are affected by chronic diseases, which represent 75% of this age group [5]. Furthermore, chronic disease prevalence is higher among rural seniors compared to their urban counterparts [6,7]. Chronic diseases not only compromise physical health but also lead to severe mental health challenges, significantly diminishing quality of life [7,8].

In the rural areas of Western China, many regions are economically underdeveloped or insufficiently developed [9,10]. Elderly populations in these areas face multiple challenges, including an incomplete medical security system, low quality of life, and a lack of spiritual support [11]. These factors compound the impact of chronic diseases on the physical and mental well-being of the elderly. Therefore, addressing the physical and mental health needs of this vulnerable group is essential to promoting healthy aging.

Subjective well-being (SWB) is a critical indicator of psychological health and life quality in older adults [12,13] and is considered an integral aspect of successful aging [14]. Previous studies have demonstrated a close association between health status and SWB, with chronic illnesses in the elderly posing a significant threat to their SWB [15,16]. Seniors with chronic diseases typically report lower levels of happiness compared to those without such conditions, suggesting that higher SWB is linked to a lower prevalence of chronic diseases [17]. Greater SWB fosters higher energy levels, which is crucial for disease management and recovery [18]. Long-term health issues often give rise to mental health problems, such as anxiety and depression, further reducing SWB and overall quality of life [14]. SWB is influenced not only by physical health but also by various factors, including mental health, social support, and healthy behaviors [15–17].

Social support, defined as the material and emotional assistance individuals receive from relationships such as family, friends, colleagues, or group organizations [19], is a key factor influencing the successful aging of elderly individuals [20] and a critical determinant of quality of life for older adults with chronic diseases [21]. Research has consistently shown that positive social support is vital for maintaining the happiness of patients with chronic diseases [22,23]. As an external resource, social support reduces life pressures for the elderly, enhancing their sense of happiness [24,25]. A study of elderly individuals in Iran revealed that both the quantity and quality of social support are significant predictors of happiness in older adults [26]. Research by Mo et al. suggests that social support mediates the relationship between mental health, physical health, and SWB [24]. Lin and Yeh assert that social support can influence cognitive evaluations, fostering a sense of care and acceptance, thereby reducing psychological stress and improving mental health [27]. A meta-analysis of studies from Western countries found that older adults with greater social support were less likely to develop depression [28], and perceived social support is inversely correlated with depressive symptoms in later life [29]. The absence of social support increases the risk of depression in the elderly [21,28]. Thus, social support plays a pivotal role in maintaining the physical and mental health of older adults [30,31].

Depression is a prevalent emotional disorder among older individuals [32], serving as a key indicator of mental health in the elderly population. Multiple meta-analyses have demonstrated that the prevalence of depressive symptoms among the global elderly population ranges from 19.2% to 31.74%[33–35]. In China, the detection rate of depression in older adults with chronic diseases ranges from 40.2% to 48.86% [36], notably higher than the global average for elderly depression. Additionally, due to the generally lower income levels in rural areas, elderly individuals face greater living pressures and psychological burdens [37], with depression rates higher in rural seniors compared to their urban counterparts [38]. Numerous studies, both domestic and international, have demonstrated a negative correlation between depression and SWB in older adults [30,39]. Some research suggests that the prolonged duration of chronic diseases, which leads to physical and social functional impairments and increased economic burdens, makes elderly individuals more susceptible to depression than their healthy counterparts, thereby significantly reducing their overall well-being [25,40]. The coexistence of depression in older adults with chronic diseases can exacerbate their health deterioration, leading to increased disability and mortality rates [35,40,41]. Furthermore, individuals struggling with depression often lack a strong social support network, highlighting the role of insufficient social support as a contributing factor to depression [42,43].

Extensive research has examined the pairwise relationships among depression, SWB, and social support in older adults [18,27–31]. However, studies explicitly investigating the triadic relationship among depression, social support, and SWB remain scarce. Existing research has primarily focused on developed regions or urban populations [17,44], with a notable lack of studies addressing elderly individuals with chronic diseases in rural areas of Western China. Qiannan, located in Guizhou Province, is an ethnically diverse, predominantly rural, and mountainous region in Western China. Due to historical factors, the area suffers from limited economic and healthcare resources, a condition shared by many rural areas in this region [11,45]. Therefore, research on the physical and mental health of elderly individuals in this region is representative of the broader rural elderly population in Western China. This study thus aims to explore the relationships between social support, depression, and SWB among elderly patients with chronic diseases living in rural Qiannan, Guizhou, with a particular focus on the mediating role of social support in the depression-SWB relationship. The goal is to provide theoretical insights for improving the physical and mental health, as well as the quality of life, of elderly populations in rural areas. Based on the literature review and research objectives, the following hypotheses were proposed:

Hypothesis 1 (H1): Depression among elderly individuals with chronic diseases in rural Western China is expected to negatively impact their SWB;

Hypothesis 2 (H2): Social support is anticipated to positively influence the SWB of elderly individuals with chronic diseases in this demographic;

Hypothesis 3 (H3): Social support is expected to act as a mediating factor in the relationship between depression and SWB in this population.

## Methods

### Ethical approval

This study received approval from the Ethics Review Committee of Qiannan Medical College for Nationalities (Approval No. 202209). Prior to completing the questionnaires, participants provided informed consent, which emphasized that participation was voluntary and that they could withdraw at any time. All procedures followed in this study were in strict accordance with the principles outlined in the Declaration of Helsinki.

### Participants

The study recruited elderly individuals aged 60 and above with chronic diseases residing in the ethnic rural regions of Qiannan, Guizhou, China, between July 20, 2022, and September 30, 2022. The inclusion criteria were: 1) residents aged 60 and above with a residence history of at least five years; 2) individuals registered with local health clinics for at least one chronic disease; and 3) willingness to provide informed consent and participate voluntarily. Exclusion criteria included: 1) severe visual or hearing impairment; 2) a history of severe mental illness; and 3) refusal to participate in the survey.

To ensure a high participation rate, the study collaborated closely with local community health service centers, which have strong connections with the elderly population. Through community meetings and health seminars, detailed explanations of the study’s purpose and potential benefits to the community were provided, emphasizing how the research could enhance healthcare services for elderly patients with chronic diseases. This approach significantly boosted participant engagement. No financial incentives or compensation were offered during recruitment. The high participation rate was attributed to the elderly population’s trust in community health service centers and their intrinsic motivation to contribute to research benefiting their peers and future generations. This strategy not only ensured a high participation rate but also minimized the risk of bias associated with financial incentives. A total of 2,201 participants completed the questionnaires; 45 were excluded due to incomplete responses, resulting in 2,156 valid responses and an effective response rate of 97.96%.

### Procedures

Elderly individuals with chronic diseases residing in the rural areas of the Qiannan ethnic region in China were included in this study. To minimize survey errors and enhance the representativeness of the sample, a stratified random cluster sampling method was employed. Initially, the 12 counties of Qiannan Prefecture were categorized into three economic levels: high, medium, and low. From each economic level, two counties were randomly selected as the first-level samples. Then, within each selected first-level sample county, two townships were randomly chosen as the second-level samples. Finally, 3–4 villages were randomly selected from each second-level sample township to serve as the third-level samples. All elderly individuals in the selected villages who met the survey criteria were included in the study.

The survey was conducted by a team of 12 investigators, consisting of licensed physicians and medical students, using a questionnaire survey method. Prior to the survey, the project leader provided training for the investigators, focusing on the survey content and standardizing the survey language. During the survey, family doctors from the selected villages provided lists of elderly individuals with chronic disease health records. All eligible elderly patients with chronic diseases from these lists were then gathered for the survey. Investigators conducted one-on-one surveys on-site, and for individuals with mobility issues, home visits were arranged for one-on-one interviews. Informed consent was obtained from all participants, and questionnaires were distributed accordingly. Elderly individuals were guided to self-complete the questionnaires, or the investigators recorded their responses based on participants’ answers. Completed questionnaires were promptly collected, and invalid questionnaires were excluded from the analysis.

### Measures tools

#### General information questionnaire.

The questionnaire was designed based on a comprehensive literature review and aligned with the study’s research objectives. It aimed to gather basic demographic and health-related information, including gender, age, ethnicity, education level, marital status, children living away from home, living arrangements, family relationships, the number and severity of chronic diseases, disease severity, self-care abilities, participation in social activities, family doctor contract services, and involvement in poverty alleviation programs such as targeted poverty alleviation and the minimum living standard guarantee system. Age was categorized into three groups: 60–70, 71–80, and over 80. Education was classified as Illiterate, Primary school, Junior high school, and High school and above. Marital status was divided into two categories: married and living with a partner, and other marital statuses (divorced, widowed, or never married). Living arrangement was classified as living alone or not living alone. Family relationship quality was assessed through self-evaluation by asking participants, “How do you think your relationship with your family members is?” Responses were grouped into four levels: Poor, Average, Quite good, and Very good. To ensure the accuracy and comprehensiveness of chronic disease information, a dual-verification method was employed, integrating self-reported data with chronic disease health records. During the initial screening, participants were asked about their history of physician-diagnosed chronic diseases using a structured questionnaire. Simultaneously, the “chronic disease health records” maintained at local community health service centers were reviewed. Cross-referencing these two data sources ensured the reliability of the chronic disease information. Only chronic diseases confirmed by both methods were included in the study. The primary diseases considered were hypertension, diabetes, cardiovascular and cerebrovascular diseases (including coronary heart disease and stroke), cervical or lumbar diseases, osteoarthritis, chronic pulmonary diseases (such as chronic bronchitis, emphysema, cor pulmonale, and asthma), and cancer. Disease severity was assessed using a 3-level rating based on both the records in chronic disease health files and the results of on-site health check-ups: Mild, Moderate, or Severe. Self-care ability was evaluated using the Basic Activities of Daily Living (BADL) scale [46], which includes six daily living abilities: bathing, dressing, eating, toileting, grooming, and walking. If all six tasks could be completed independently, the individual was classified as fully self-care capable. If 1–3 tasks could not be performed independently, the individual was considered partially self-care capable. If 4 or more tasks could not be completed independently, the individual was classified as incapable of self-care. Social activity participation was assessed by asking, “Over the past month, how often have you participated in social activities such as playing cards, chess, mahjong, square dancing, singing, or shopping?” Responses were categorized as follows: Always participate (almost every day), Sometimes participate (at least once a week), Occasionally participate (at least once a month), and Never participate (no participation in the past month).

#### Short Form Geriatric Depression Scale (GDS-15).

The GDS-15, developed by Yesavage et al. [47], is designed to assess depressive symptoms in participants. The scale includes 15 items, scored with 1 point for “yes” and 0 for “no,” with some items reverse-scored. Total scores range from 0 to 15, classifying depression levels as follows: no symptoms (0–5), mild symptoms (6–10), and moderate to severe symptoms (11–15) [48]. The Cronbach’s α coefficient for this scale in the study was 0.797, reflecting acceptable internal consistency.

#### Social Support Rating Scale (SSRS).

The SSRS, developed by Xiao S Y [49], is commonly used to assess individuals’ social support levels. The scale consists of 10 items across three dimensions: objective support, subjective support, and utilization of support. (1) Objective Support Score: This score is the sum of items 2, 6, and 7. Items 6 and 7 are scored based on the number of support sources identified by the participant, with “no source” receiving a score of 0. For instance, if a participant identifies three support sources for item 6, the score for that item is 3; (2) Subjective Support Score: Calculated from the sum of items 1, 3, 4, and 5. Item 5 includes four sub-items (A, B, C, and D), each rated on a 1–4 scale (1 = no support, 4 = full support), with the sub-item scores combined to form the total for item 5; (3) Utilization of Support Score: This score is obtained by summing items 8, 9, and 10, each rated on a 4-point scale (1 = no support, 4 = adequate support). The combined score reflects the participant’s utilization of available support. The total social support score, ranging from 12 to 66, is the sum of all 10 items, with higher scores indicating stronger social support. For this study, social support was categorized as high (≥ 45), moderate (23–44), or low (< 23). The Cronbach’s α coefficient for this scale in the study was 0.765, reflecting acceptable internal consistency.

#### Memorial University of Newfoundland Scale of Happiness (MUNSH).

The MUNSH, developed by Kozma et al. [50], is used to assess patients’ SWB. This scale comprises 24 items across four component scales: Positive Affect (PA), Negative Affect (NA), Positive Experience (PE), and Negative Experience (NE). PA and NA represent emotional dimensions, while PE and NE reflect experience and perception dimensions. PA and NA each contain 5 items, while PE and NE each contain 7 items. Responses are scored as follows: “yes” = 2 points, “don’t know” = 1 point, and “no” = 0 points. Specific scoring is applied to certain items: Item 19, asking about preferred residence, scores 2 points for “current residence” and 0 points for “other places”; Item 23, asking about life satisfaction, scores 2 points for “satisfied” and 0 points for “not satisfied.” The total MUNSH score is calculated as PA score – NA score + PE score – NE score + 24, with a range from 0 to 48. SWB levels are classified as high (≥ 36), moderate (13–35), or low (≤ 12), with lower scores indicating lower SWB. The Cronbach’s α coefficient for this scale in the study was 0.811, indicating acceptable internal consistency.

### Data analysis

Data entry was performed using Epidata 3.1 software, incorporating a dual-check process to ensure accuracy. Statistical analysis was conducted with SPSS 23.0, considering two-sided *P*-values < 0.05 as statistically significant. Categorical data were presented as counts (n) and percentages (%), while continuous data were expressed as means ± standard deviations. Group comparisons for continuous variables were performed using t-tests, and *t/F*-tests were used for categorical variables. Pearson correlation and hierarchical linear regression analyses examined the relationships between depression, social support, and SWB. To test the mediating role of social support in the relationship between depression and SWB, structural equation modeling (SEM) was performed using Amos 23.0. Model fit was evaluated using several indices, including the goodness of fit index (GFI), Normed Fit Index (NFI), Relative Fit Index (RFI), incremental fit index (IFI), Tucker-Lewis index (TLI), comparative fit index (CFI), and root mean square error of approximation (RMSEA). Acceptable model fit was defined as GFI, NFI, RFI, IFI, TLI, and CFI values > 0.9 and RMSEA < 0.08 [51]. The percentile bootstrap method with 5000 samples and bias-corrected 95% confidence intervals (CIs) was employed. Mediation effects were considered present if the CI did not include zero [51,52].

## Results

### Characteristics of demographic variables and differences in subjective well-being

Among the 2,156 participants included in the analysis, 1,104 were male (51.2%). The mean age was 71.15 ± 8.04 years, with 1,182 individuals (54.8%) aged 60–70, 630 individuals (29.2%) aged 71–80, and 344 individuals (16.0%) aged over 80. Regarding ethnicity, 1,416 individuals (65.7%) belonged to minority ethnic groups. In terms of education, 1,168 participants (54.2%) were illiterate, 702 (32.6%) had completed primary school, 208 (9.6%) had completed junior high school, and 78 (3.6%) had completed high school or higher education. There were 1,476 individuals (68.5%) who were married and living with their partner, while 680 individuals (31.5%) had other marital statuses (divorced, widowed, or never-married).

Univariate analysis revealed significant differences in MUNSH scores across various demographic factors, including education, marital status, children working away from home, living arrangement, family relationships, disease severity, self-care ability, social activity participation, family doctor contracts, targeted poverty alleviation households, and minimum living allowance (all *P* < 0.05). Detailed results are presented in Table 1.

**Table 1:** Descriptive statistics of demographic variables and differences in SWB (N = 2156).

Variable	Category	*N (%)*	MUNSH score (M ± SD)	*t/F*	*P*
Gender	Female	1052(48.8)	27.00 ± 9.70	−0.829	0.407
	Male	1104(51.2)	27.34 ± 9.26		
Age (years)	60-70	1182(54.8)	27.31 ± 9.48	0.299	0.742
	71-80	630(29.2)	27.07 ± 8.91		
	>80	344(16.0)	26.90 ± 10.44		
Nation	Han nationality	740(34.3)	27.21 ± 9.23	0.123	0.902
	Ethnic Minorities	1416(65.7)	27.16 ± 9.61		
Education	Illiterate	1168(54.2)	26.65 ± 9.71	4.042	0.007
	primary school	702(32.6)	27.44 ± 8.96		
	Junior high school	208(9.6)	28.99 ± 10.09		
	High school and above	78(3.6)	27.80 ± 8.08		
Marital status	Other marital status (divorced, widowed, and never-married)	680(31.5)	26.03 ± 9.41	−3.807	<0.001
	Married and with a surviving partner	1476(68.5)	27.70 ± 9.47		
Children working away from home	No	894(41.5)	28.51 ± 9.500	4.800	<0.001
	Yes	1262(58.5)	26.35 ± 9.389		
Living arrangement	Living alone	352(16.3)	25.58 ± 10.07	−3.459	0.001
	Not living alone	1804(83.7)	27.48 ± 9.33		
Family relationships	Poor	66(3.1)	18.00 ± 6.37	−172.352	<0.001
	Average	402(18.6)	22.74 ± 7.78		
	Quite good	854(39.6)	25.13 ± 9.33		
	Very good	834(38.7)	32.13 ± 8.11		
Number of chronic diseases (types)	1	1120(51.9)	27.08 ± 9.36	2.089	0.124
	2	662(30.7)	26.84 ± 8.74		
	≥3	374(17.4)	28.05 ± 10.94		
Disease severity	Mild	712(33.0)	29.29 ± 9.45	27.144	<0.001
	Moderate	1110(51.5)	26.12 ± 9.22		
	Severe	334(15.5)	26.15 ± 9.65		
Self-care ability	Unable to self-care	134(6.2)	23.30 ± 12.00	−30.491	<0.001
	Partial self-care	826(38.3)	25.92 ± 9.63		
	Complete self-care	1196(55.5)	28.47 ± 8.80		
Social Activity Participation Status	Never participate	453(21.0)	25.00 ± 9.94	−38.516	<0.001
	Occasionally participate	1128(52.3)	26.51 ± 9.02		
	Sometimes participate	347(16.1)	28.69 ± 9.63		
	Always participate	228(10.6)	32.48 ± 8.16		
Family doctor contract	No	152(7.1)	20.83 ± 6.08	−12.703	<0.001
	Yes	2004(92.9)	27.66 ± 9.52		
targeted poverty alleviation households	No	924(42.9)	21.85 ± 8.20	−25.922	<0.001
	Yes	1232(57.1)	31.17 ± 8.34		
Minimum living allowance	No	1718(79.7)	27.58 ± 9.66	4.297	<0.001
	Yes	438(20.3)	25.57 ± 8.53		

Note: SWB: Subjective Well-being; MUNSH: the Memorial University of Newfoundland Happiness Scale

### Descriptive statistics of depression, social support, and subjective well-being

Of the participants, 512 (23.7%) had high SWB, 1,538 (71.3%) had moderate SWB, and 106 (4.9%) had low SWB. Regarding depressive symptoms, 627 (29.1%) had no depressive symptoms, 1,246 (57.8%) had mild depressive symptoms, and 283 (13.1%) had moderate to severe depressive symptoms. In terms of social support, 102 (4.7%) had low social support, 1,786 (82.8%) had moderate social support, and 268 (12.4%) had high social support.

The total MUNSH score for elderly patients with chronic diseases was 27.17 ± 9.48, with PA at 5.02 ± 2.64, NA at 4.06 ± 3.00, PE at 7.56 ± 3.60, and NE at 5.35 ± 3.67. The GDS-15 score was 7.23 ± 2.59. The SSRS total average score was 34.96 ± 7.97, with subjective support at 18.80 ± 4.21, objective support at 8.63 ± 3.47, and support utilization at 7.53 ± 2.04. Detailed results are provided in Table 2.

**Table 2:** Descriptive statistics of the main variables (N = 2156).

Variable	Mean	SD	Range
GDS-15 score	7.23	2.59	2–14
SSRS score	34.96	7.97	16–57
Subjective support	18.8	4.21	8–28
Objective support	8.63	3.47	1–18
Support utilization	7.53	2.04	3–12
MUNSH score	27.17	9.48	4–46
PA	5.02	2.64	0–10
NA	4.06	3.00	0–10
PE	7.56	3.60	0–14
NE	5.35	3.67	0–14

Note: GDS-15: Geriatric Depression Scale; SSRS: the Social Support Rating Scale; MUNSH: the Memorial University of Newfoundland Happiness Scale; PA: positive affect; NA: negative affect; PE: positive experiences; NE: negative experiences.

### Correlational analysis of the main variables

Pearson correlation analysis revealed that the GDS-15 score was negatively correlated with MUNSH, PA, and NA scores (*r* = −0.528, −0.139, −0.276, *P* < 0.01), and positively correlated with PE and NE scores (*r* = 0.546, 0.546, *P* < 0.05). The SSRS total score, subjective support score, objective support score, and support utilization score were all negatively correlated with the GDS-15 score (*r* = −0.243, −0.256, −0.177, −0.121, *P* < 0.05). The SSRS total score was positively correlated with MUNSH, PA, and NA scores (*r* = 0.280, 0.282, 0.173, *P* < 0.05), and negatively correlated with PE and NE scores (*r* = −0.245, −0.149, *P* < 0.05). Subjective support was positively correlated with MUNSH, PA, and NA scores (*r* = 0.258, 0.242, 0.124, *P* < 0.05), and negatively correlated with PE and NE scores (*r* = −0.222, −0.189, *P* < 0.05). Objective support showed positive correlations with MUNSH, PA, and NA scores (*r* = 0.204, 0.199, 0.170, *P* < 0.05), and negative correlations with PE and NE scores (*r* = −0.177, −0.071, *P* < 0.05). The support utilization score was positively correlated with MUNSH, PA, and NA scores (*r* = 0.214, 0.264, 0.132, *P* < 0.05), and negatively correlated with PE and NE scores (*r* = −0.197, −0.073, *P* < 0.05). Detailed results are provided in Table 3.

**Table 3:** Correlational analysis of the main variables (N = 2156).

Variable	GDS-15 score	SSRS score	Subjective support	Objective support	Support utilization	MUNSH score	PA	NA	PE	NE
GDS-15 score	1									
SSRS score	−0.243^**^	1								
Subjective support	−0.256^**^	0.878**	1							
Objective support	−0.177^**^	0.815**	0.509**	1						
Support utilization	−0.121^**^	0.708**	0.500**	0.432**	1					
MUNSH score	−0.528^**^	0.280**	0.258**	0.204**	0.214**	1				
PA	−0.139^**^	0.282**	0.242**	0.199**	0.264**	0.656**	1			
NA	0.546^**^	−0.245**	−0.222**	−0.177**	−0.197**	−0.778**	−0.316**	1		
PE	−0.276^**^	0.173**	0.124**	0.170**	0.132**	0.737**	0.572**	−0.289**	1	
NE	0.546^**^	−0.149**	−0.189**	−0.071**	−0.073**	−0.750**	−0.155**	0.681**	−0.274**	1

Note: GDS-15: Geriatric Depression Scale; SSRS: the Social Support Rating Scale; MUNSH: the Memorial University of Newfoundland Happiness Scale; PA: positive affect; NA: negative affect; PE: positive experiences; NE: negative experiences;* * represents *P* < 0.01.

### Hierarchical regression analysis

Based on the univariate and correlation analyses, hierarchical regression analysis was performed to explore the associations among key variables. SWB was the dependent variable, while depression and social support were the independent variables. Demographic variables that were statistically significant in the univariate analysis were included as control variables. Model 1 included only the demographic control variables; Model 2 added the depression variable; and Model 3 further incorporated social support. The results showed that all models were statistically significant (*P* < 0.001). Model 2 revealed that depression had a significant negative effect on SWB (*β* = −0.462, *P* < 0.001). In Model 3, both depression (*β* = −0.455, *P* < 0.001) and social support (*β *= 0.094, *P* < 0.001) significantly influenced SWB levels, after controlling for demographic variables. Notably, the negative effect of depression on SWB diminished with the inclusion of social support, suggesting a partial mediating role of social support between depression and SWB. Detailed results are presented in Table 4.

**Table 4:** Hierarchical regression analysis of SWB.

Variable		Model1		Model2		Model3	
		*β*	*t*	*β*	*t*	*β*	*t*
	Depression			−0.462	−29.476***	−0.455	−29.005***
	Social Support					0.094	4.501***
Education	Illiterate	Ref					
	primary school	0.054	2.9**	0.073	4.696***	0.08	5.106***
	Junior high school	0.04	2.239*	0.061	4.021***	0.065	4.288***
	High school and above	0.022	1.245	0.014	0.957	0.017	1.122
Marital status	Other marital status (divorced, widowed, and never-married people)	Ref					
	Married and with a surviving partner	0.023	1.281	−0.007	−0.492	−0.015	−0.99
Children working away from home	No	Ref					
	Yes	−0.048	−2.667**	−0.005	−0.351	−0.012	−0.779
Living arrangement	Living alone	Ref					
	Not living alone	0.006	0.338	−0.075	−4.875***	−0.114	−6.483
Family relationships	Poor	Ref					
	Average	0.214	5.174***	0.239	6.837***	0.224	6.427
	Quite good	0.331	6.616***	0.314	7.447***	0.292	6.912
	Very good	0.574	11.339***	0.517	12.105***	0.489	11.366
Disease severity	mild	Ref					
	Moderate	−0.036	−1.802	−0.044	−2.662**	−0.045	−2.715
	Severe	−0.055	−2.763**	−0.06	−3.559***	−0.061	−3.638***
Self-care ability	Unable to self-care	Ref					
	Partial self-care	0.061	1.564	0.052	1.562	0.059	1.787
	Complete self-care	0.101	2.545*	0.07	2.076**	0.064	1.927
Social Activity Participation Status	Never participate	Ref					
	Occasionally participate	0.05	2.067*	0.078	3.767***	0.055	2.628***
	Sometimes participate	0.117	5.278***	0.076	4.039***	0.047	2.392***
	Always participate	0.126	5.939***	0.151	8.451***	0.106	5.167***
Family doctor contract	No	Ref					
	Yes	0.055	2.961**	−0.022	−1.377	−0.031	−1.972*
targeted poverty alleviation households	No	Ref					
	Yes	0.373	20.01***	0.361	22.953***	0.363	23.195***
Minimum living allowance	No	Ref					
	Yes	−0.046	−2.545*	0.02	1.265	0.019	1.256
	*R2*	0.385		0.563		0.567	
	*△R2*	0.385		0.178		0.004	
	*F*	70.41***		137.51***		133.108***	

Note: SWB: Subjective Well-being; Significance: * *p* < 0.05, **p < 0.01, ***P < 0.001. Dependent variables: subjective well-being. Independent variable: Model 1: demographic control variables; Model 2: demographic control variables and depression variable; Model 3: demographic control variables, depression variable, and social support variable.

### Mediation analysis

To further investigate the mediating role of social support in the relationship between depression and SWB among older adults with chronic diseases, an SEM was constructed using AMOS software. The fit indices of the structural model were calculated to assess the alignment with the theoretical model. The SEM analysis yielded satisfactory fit indices: χ²/df = 3.836, GFI = 0.994, NFI = 0.987, RFI = 0.967, IFI = 0.989, TLI = 0.971, CFI = 0.989, RMSEA = 0.056, indicating that the model achieved a good fit. The path coefficients revealed that depressive symptoms significantly negatively impacted both social support (path coefficient = −0.28) and SWB (path coefficient = −0.47) among older adults with chronic diseases.

**Fig 1:**
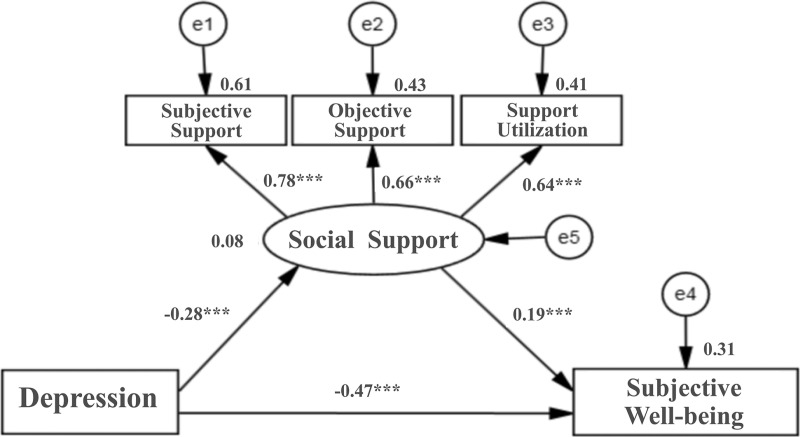
Mediation model of the effect of social support on depression and subjective well-being. **Note:** Factor loadings are standardized; significant paths are indicated by asterisks (*** *P* < 0.001).

Conversely, social support had a significant positive effect on SWB (path coefficient = 0.19). All path coefficients were statistically significant (all *P* < 0.001), confirming the strength of the relationships in the model. The specific structural model is illustrated in Fig 1.

Subsequently, a bootstrap test with 5000 resamples was performed. The bootstrap test results showed that the total effect of depressive symptoms on SWB in older adults with chronic diseases was −0.528 (95% *CI*: −0.553 to −0.502, *P* < 0.001), with a direct effect of −0.474 (95% *CI*: −0.503 to −0.443, *P* < 0.001), accounting for 89.77% of the total effect. The mediating effect of social support in the relationship between depression and SWB was −0.133 (95% *CI*: −0.070 to −0.041, *P* < 0.001), indicating that social support partially mediates the relationship between depression and SWB, with the mediating effect accounting for 10.23% of the total effect. Detailed results of the bootstrap mediation analysis are presented in Table 5.

**Table 5:** Bootstrap test and effect size of the mediation effect of social support on depression and SWB (Standardized coefficients).

Effect	Path	Effect value	Effect ratio (%)	95%*CI*^a^		*P* value
				Lower bonds	Up bonds	
Direct effect	Depression→subjective well-being	−0.474	89.77%	−0.503	−0.443	0.000***
Mediation effect	Depression → social support →SWB	−0.133	10.23%	−0.070	−0.041	0.000***
Total effect		−0.528	100%	−0.553	−0.502	0.000***

Note: SWB: Subjective Well-being; Significance: ^a^ represents the 95% confidence interval calculated in percentage form; CI: confidence interval standardized estimating of 5000 bootstrap sample: p*** < 0.001

## Discussion

This study explored the intricate relationships between depression, social support, and SWB in elderly patients with chronic diseases living in rural areas of Western China, and validated the mediating role of social support in the relationship between depression and SWB through SEM. The findings suggest that SWB levels among elderly patients in this region are relatively low, with a high prevalence of depressive symptoms. Depression is significantly negatively correlated with SWB, while social support is significantly positively correlated with SWB. Furthermore, social support partially mediates the relationship between depression and SWB.

### Analysis of the current status of subjective well-being, depression, and social support

The level of SWB in elderly patients with chronic diseases in rural Western China is moderately low, even lower than that observed in elderly patients in other rural regions, as reported by Dong et al [53]. This reflects the severe health and well-being challenges faced by this population. Contributing factors may include the environmental and economic conditions typical of rural Western China, as well as the high prevalence of comorbidities and disease severity. Rural Western China is characterized by geographical isolation, underdeveloped economies, and limited access to medical insurance systems [11,45], all of which create multidimensional barriers to the physical and mental health, as well as the overall quality of life, of elderly patients with chronic diseases. Both domestic and international studies have established a clear link between the high incidence and severity of chronic diseases and the decline in mental health and quality of life [54,55]. In the current study, 48.1% of elderly patients had multiple chronic conditions, and 67% experienced moderate to severe disease. The high prevalence and severity of these chronic diseases likely contribute to increased physical, psychological, and financial stress, exacerbating mental health issues such as depression and anxiety [56,57]. These factors are closely tied to the lower SWB observed in this population. Furthermore, economic pressures result in 58.5% of patients’ adult children working away from home for long periods, diminishing family companionship, daily care, and emotional support [55], which further adversely impacts their SWB.

This study revealed that the overall level of depressive symptoms among elderly patients with chronic diseases in rural areas of Western China is moderate, with a depression detection rate of 70.9%. This proportion is significantly higher than that reported by Chang and Wu [36,58], underscoring the severity of depression in this population. Contributing factors may include: First, chronic diseases, as persistent stressors, repeatedly activate neurons, leading to unstable neural activity, which may increase individuals’ susceptibility to depression [59]. Second, the long-term burden of chronic diseases not only exacerbates the economic strain on families but also increases caregiving responsibilities, placing significant psychological stress on elderly patients. This may foster feelings of helplessness and despair [60], thus elevating the risk of depressive symptoms. Third, the generally lower educational attainment of elderly individuals in rural areas may hinder their understanding of health and psychological issues and their ability to cope with them [11]. Additionally, the relatively low accessibility of medical and mental health services in these areas further restricts opportunities for elderly patients with chronic diseases to seek help, potentially exacerbating depressive symptoms [37].

The study also indicated that social support levels among elderly patients with chronic diseases in rural Western China are moderate, slightly higher than those reported by Cao et al [61]. in their study of similar populations. Several factors may explain this: On one hand, recent government efforts to support rural areas, particularly those with ethnic minorities, through policies such as targeted poverty alleviation and family doctor contracting have provided direct economic, medical, and health management support to elderly individuals [62–64]. These initiatives have positively influenced their levels of social support. On the other hand, the chronic and complex nature of long-term illnesses requires significant human and financial resources from families to manage [65]. However, the limited economic resources in rural areas often increase psychological stress. This stress can negatively impact psychological well-being, which, in turn, may limit the acquisition and effective utilization of social support [66], potentially restricting the overall level of social support available to this group.

### The impact of depression on subjective well-being

In this study, a significant negative correlation was observed between depression and SWB, with more severe depressive symptoms associated with lower SWB. This finding aligns with previous literature [67,68], emphasizing the strong connection between depression and diminished SWB in elderly patients with chronic diseases in rural areas. Several contributing factors may explain this relationship: First, persistent depressive symptoms may impact the autonomic nervous system’s physiological functions, potentially leading to cognitive impairment in the elderly by reducing brain-derived neurotrophic factor (BDNF) activity and altering brain structure [69]. These physiological changes may contribute to the negative effects of depression on SWB. Second, individuals with depression typically exhibit lower psychological resilience. Elderly patients with reduced psychological resilience may lack effective coping strategies when confronted with the long-term stressors associated with chronic illnesses, leading to feelings of helplessness and despair [70]. This inability to cope effectively may hinder their positive assessment of life. Third, depressive moods can impair emotional regulation, making it difficult for elderly patients to recover from negative emotions [71]. This lack of emotional regulation can cause the accumulation and worsening of negative emotions, which in turn negatively affects their SWB.

Further correlation analyses revealed significant positive associations between depression and negative emotions, as well as negative experiences, in elderly patients with chronic diseases. In contrast, significant negative associations were found with depression and positive emotions, as well as positive experiences. Some studies suggest that elderly patients with chronic diseases who experience depressive symptoms may feel hopeless and lack purpose in life [72]. They often endure significant long-term psychological stress, making them more vulnerable to negative emotions such as self-blame, inferiority, and physical discomfort. This emotional burden can lead to a decline in social functioning, reduced energy levels, and decreased satisfaction and interest in life, thereby diminishing positive life experiences [73]. Prolonged negative self-evaluation and emotional distress may decrease their motivation and initiative to engage in various activities, resulting in increased social isolation and withdrawal from social interactions [43]. This reduction in social engagement can limit opportunities to receive social support, further hindering the maintenance of positive emotions and experiences.

### Impact of social support on subjective well-being

This study emphasizes the significant positive effect of social support on SWB, further validating the direct effect model of social support [74]. This highlights the critical role social support plays in improving the quality of life for elderly patients with chronic diseases. Social support enhances SWB through several mechanisms. First, it creates a more positive social environment, providing elderly patients with a sense of belonging and security [60]. This sense of security fosters greater hope, contributing to overall improvements in SWB [21]. Additionally, social support strengthens feelings of self-efficacy and satisfaction, which in turn elevate self-esteem [75]. This boost in self-esteem is conducive to further enhancing SWB. Cohen and Wills [76] have shown that social support increases positive emotional experiences, life predictability, and self-worth, all of which contribute to the positive impact of social support on the SWB of elderly patients with chronic diseases.

Social support exerts a more substantial effect on the emotional dimension of SWB than on the experiential dimension. This difference may stem from the direct emotional regulation that social support provides. Emotional comfort and practical assistance can quickly improve emotional states, resulting in more noticeable and immediate changes in this dimension [77]. In contrast, the experiential dimension, influenced by personal values, life goals, and social comparisons, tends to change more gradually and in a more complex manner [21]. This suggests that interventions aimed at enhancing the SWB of elderly individuals in rural areas should prioritize emotional support to achieve more immediate and significant improvements.

### The mediating role of social support

The mediating effect analysis in this study revealed that social support acts as a partial mediator between depression and SWB. This suggests that depression not only directly affects the SWB of elderly patients with chronic diseases in rural areas but also indirectly influences their SWB through social support. Higher levels of social support can buffer the negative impact of depression on well-being, further corroborating the buffering effect of social support [74,78]. Consistent with the buffering model of social support [78], social support has been shown to alleviate psychological stress, reduce the negative emotional experiences associated with stress, and enhance positive emotional experiences, which in turn help lower the incidence of depression [70,74]. Empirical studies indicate that social support strengthens the functioning of neuroendocrine and immune systems, improves cognitive function, boosts positive emotions, and diminishes negative emotions [79,80]. Furthermore, robust social support can enhance psychological resilience and hardiness in the elderly. High psychological resilience enhances an individual’s ability to cope with stress and mitigates the detrimental impact of depressive symptoms on SWB [70].

Pearson’s analysis further revealed that among the three dimensions of social support—subjective, objective, and utilization—subjective support had the greatest influence on both SWB and depression. This finding differs from that of Pang et al. [81], but aligns more closely with the results of Su et al [82]. This supports the idea that emotional and perceived support plays a more critical role in influencing mental health and overall well-being than tangible forms of support [81,83]. Subjective support, primarily involving psychological and emotional assistance, contrasts with objective support, which includes tangible resources such as financial aid and policy support [44]. Recent policy initiatives, including precision poverty alleviation and family doctor programs, have increased objective support for elderly patients with chronic diseases in rural areas, addressing material needs while also highlighting psychological needs [62,84]. According to life course theory, as older individuals experience physical decline and social role changes, their demand for psychological comfort intensifies [85]. Adequate emotional support can enhance their positive emotions, strengthen their confidence in managing illness, foster a sense of belonging and purpose, and alleviate depressive symptoms, thereby improving SWB [14]. These findings underscore the critical role of emotional support in enhancing the SWB of elderly patients with chronic diseases in rural Western China. Enhancing social support, particularly emotional support, is thus an effective strategy to improve their SWB.

### Strength and limitations

This study integrated both physical and mental health factors, positioning social support as a critical mediator between depression and SWB. It provides a comprehensive exploration of the mechanisms influencing SWB among elderly patients with chronic diseases in rural areas. The findings highlight the essential role of social support for the elderly, suggesting that prioritizing social support alongside physical and mental health care may be a key strategy for enhancing overall well-being. However, several limitations exist. First, the survey data are geographically restricted, and the sample size is relatively small. Second, biases in self-reported data could affect the reliability of the results. Third, as a cross-sectional study, the ability to draw definitive causal conclusions is limited. Future studies will aim to broaden the research scope, increase the sample size, incorporate additional variables related to SWB, and conduct longitudinal studies to provide more robust evidence for the conclusions of this study.

## Conclusion

In conclusion, this study utilized SEM to examine the psychological and social mechanisms influencing the SWB of elderly patients with chronic diseases in rural areas of Western China. The findings underscore the significance of social support, particularly subjective support, in reducing depressive symptoms and improving SWB in this population. To enhance the SWB of elderly patients with chronic diseases in rural areas, collaborative efforts from national and local governments, communities, and families are necessary to strengthen the social support system across various domains, particularly by increasing psychological and emotional support. Such initiatives are crucial for improving the mental health and SWB of elderly individuals in rural areas and provide a foundation for developing targeted interventions and policies.

## Supporting information

S1 Appendix Full Survey Questionnaires Related to the Manuscript (DOCX)

S1 Dataset The original dataset of the survey related to this study. (XLSX)
