# Supplementary material for: Social support mediates the relationship between depression and subjective well-being in elderly patients with chronic diseases: Evidence from a survey in Rural Western China
Source: PLoS One. 2025 Jun 2;20(6):e0325029. doi: 10.1371/journal.pone.0325029 (PMC12129150; doi:10.1371/journal.pone.0325029)
Supplement: S1 Appendix — (DOCX) [file pone.0325029.s001.docx]

**General information questionnaire of the Elderly in Rural Areas In rural areas of Western China**

**Survey Location: Survey Date:**

**Instructions: Hello! In order to better understand your health status, we would like to ask some questions about your life and hope to receive your cooperation, thank you!**

**I. Basic Information**

1.Your Name: 2. Gender: Male  Female 3. Age (years):

4.Your Ethnicity: Han Chinese Ethnic Minority (Buyi/Shui/Miao/Yao/Maonan Dong/Others: )

5.Education Level: Illiterate Primary School

Junior High School High School and above

1. Marital Status: Married and with asurviving partner

Other marital status (divorced, widowed, and never-married)

7.Number of Children: Have children ( Sons ; Daughters ) No children

8.Are your children filial: Very filial Fairly filial Average filial Unfilial

9.Do your children work outside: Yes ( months/year) No

10.Living arrangement: Living alone ;Not living alone (with spouse, with children, with grandchildren)

11.Family relationship: Very good Fairly good Average Poor

12.Monthly Income per Capita (RMB): <1000 1001-2000 2000-3000 >3000

**II. Living and Health Conditions**

1.Self-care Ability（How many of the following six activities of daily living can you complete independently:bathing, dressing, eating, toileting, grooming, and walking）:

Fully self-sufficient all 6 items ；artially self-sufficient 1 to 3 items cannot be completed ；Unable to take care of oneself 4 or more items cannot be completed

1. Social Activity Participation Status（Over the past month, how often have you participated in social activities such as playing cards, playing chess, mahjong, square dancing, singing, or shopping? ）:

Never participate never in the past month Occasionally participate at least once a month

Sometimes participate at least once a week Always participate almost every day

3.Self-assessed Health Status: Good Average Poor

4.Number of Chronic Diseases:

4.1 Diseases suffered from: Hypertension Diabetes Coronary heart disease Stroke Rheumatoid arthritis Neck, lumbar spine diseases Chronic lung diseases (Chronic bronchitis/Emphysema/Cor pulmonale);

Cancer ： Others (if not in the list):

5.Disease Severity: Mild Moderate Severe Extremely severe

**III. Medical and Support Policies**

1.Family Doctor Contract: Yes No

2. Satisfaction with Family Doctor Contract: Very satisfied Fairly satisfied Unsatisfied

3.Are you a Poverty Alleviation Target households : Yes No

4. Are you a minimum living allowance households : Yes No

**Memorial University of Newfoundland Happiness Scale (MUNSH)**

**Instructions: Hello! We would like to ask some questions about how your life is going. Have you experienced the following feelings in the past few months? If it applies to you, please mark the corresponding column with a "√". (Note: Scoring is "Yes" = 1 point; "No" = 2 points; "Don't know" = 3 points).**

| **Item** | **Yes** | **No** | **Don't know** |
| --- | --- | --- | --- |
| 1、  Are you at your peak? |  |  |  |
| 2、 Are you in a good mood? |  |  |  |
| 3、Are you particularly satisfied with your life? |  |  |  |
| 4、 Do you feel lucky? |  |  |  |
| 5、 Are you troubled? |  |  |  |
| 6、 Do you feel very lonely or alienated from others? |  |  |  |
| 7、Are you worried or very unhappy? |  |  |  |
| 8、Do you worry because you don't know what will happen? |  |  |  |
| 9、 Do you feel resentful about your current life situation? |  |  |  |
| 10、 Overall, has your life situation improved to your satisfaction? |  |  |  |
| 11、 Is this period the most difficult time in your life? |  |  |  |
| 12、Are you as happy as you were when you were young? |  |  |  |
| 13、Are most of the things you do monotonous or boring to you? |  |  |  |
| 14、Are you still enjoying the things you were interested in the past? |  |  |  |
| 15、When you look back on your life, are you quite satisfied? |  |  |  |
| 16、As you age, are things getting worse? |  |  |  |
| 17、Do you feel very lonely? |  |  |  |
| 18、Have small things this year troubled you? |  |  |  |
| 19、If you could choose your own place to live, where would you like to choose? | Current residence | Elsewhere | Don't know |
| 20、Sometimes do you feel life is meaningless? |  |  |  |
| 21、Are you as happy now as you were when you were young? |  |  |  |
| 22、Most of the time do you feel life is hard? |  |  |  |
| 23、Are you satisfied with your current life? |  |  |  |
| 24、Compared with your peers, is your health about the same or even better? |  |  |  |

**Note:** 1. Please mark "√" on the option that fits you; for items with " ", please fill in more details;

This survey includes two forms: "General Survey of the Elderly" and "Memorial University of Newfoundland Happiness Scale". Please print on both sides and fill out completely according to the relevant requirements!

### **Detailed Description of the Memorial University of Newfoundland Scale of Happiness (MUNSH)**

#### **Introduction to the Scale**

The Memorial University of Newfoundland Scale of Happiness (MUNSH) was developed by Kozma and Stones in 1980 to assess individual happiness. The scale is based on the emotional balance theory, which views happiness as the balance between positive and negative emotions.

#### **Structure of the Scale**

The MUNSH scale consists of 24 items, divided into four main dimensions:

****Positive Affect (PA)****: Item 1 + Item 2 + Item 3 + Item 4 + Item 10

****Negative Affect (NA)****: Item 5 + Item 6 + Item 7 + Item 8 + Item 9

****Positive Experience (PE)****: Item 12 + Item 14 + Item 15 + Item 19 + Item 21 + Item 23 + Item 24

****Negative Experience (NE)****: Item 11 + Item 13 + Item 16 + Item 17 + Item 18 + Item 20 + Item 22

#### **Scoring Method**

****Scoring Criteria****: Each item is scored on a 3-point scale, with "yes" scored as 2 points, "not sure" or "don't know" scored as 1 point, and "no" scored as 0 points.

****Special Cases****:

****Item 19 (Satisfaction with current living situation)****: "Current residence" is scored as 2 points, while "other place" is scored as 0 points.

****Item 23 (Life satisfaction)****: "Satisfied" is scored as 2 points, while "unsatisfied" is scored as 0 points.

****Total Score Calculation****:

****Positive Affect Score (PA)****: Sum of all positive affect items.

****Negative Affect Score (NA)****: Sum of all negative affect items.

****Positive Experience Score (PE)****: Sum of all positive experience items.

****Negative Experience Score (NE)****: Sum of all negative experience items.

****Total Happiness Score****: Total score = PA - NA + PE - NE + 24.

#### **Total Score Classification**

According to the total score calculation formula of the MUNSH scale, the final score ranges from 0 to 48 points:

****0-12 points****: Low happiness, which may indicate higher psychological stress or emotional problems in the individual.

****13-35 points****: Moderate happiness, indicating a relative balance between positive and negative emotions and experiences.

****36-48 points****: High happiness, indicating higher life satisfaction and positive emotional experiences.

#### **Reliability and Validity of the Scale**

****Reliability****: The MUNSH scale has high internal consistency, with a Cronbach's α coefficient typically ranging from 0.70 to 0.80.

****Validity****: The scale demonstrates good validity across different cultural backgrounds and age groups.

**References**

1. Kozma A, Stones MJ. The measurement of happiness: development of the Memorial University of Newfoundland Scale of Happiness (MUNSH). J Gerontol. 1980;35(6):906-12. [https://doi.org/10.1093/geronj/35.6.906](https://doi.org/10.1093/geronj/35.6.906).
2. Zhang C, Dong F, Zheng X, et al. The impact of sleep quality on subjective wellbeing among older adults with multimorbidity: a moderated mediation model. Front Psychol. 2022;13:813775. [https://doi.org/10.3389/fpsyg.2022.813775](https://doi.org/10.3389/fpsyg.2022.813775).
3. Zhang Q, Yang Y, Zhang GL. Influence of life meaning on subjective well-being of older people: serial multiple mediation of exercise identification and amount of exercise. Front Public Health. 2021;9:515484. [https://doi.org/10.3389/fpubh.2021.515484](https://doi.org/10.3389/fpubh.2021.515484).
4. Zhou Y, Zhou L, Fu C, et al. Socio-economic factors related with the subjective well-being of the rural elderly people living independently in China. Int J Equity Health. 2015;14(1):5. [https://doi.org/10.1186/s12939-015-0136-4](https://doi.org/10.1186/s12939-015-0136-4).
5. Elavsky S, McAuley E, Motl RW, et al. Physical activity enhances long-term quality of life in older adults: efficacy, esteem, and affective influences. Ann Behav Med. 2005;30(2):138-45. [https://doi.org/10.1207/s15324796a](https://doi.org/10.1207/s15324796a) bm3002_6.
6. Chen, Y., Wang, D., Chen, W., et al. Social capital, health status, and sociodemographic factors associated with subjective well-being among older adults: a comparative study of community dwellings and nursing homes. BMC public health, 2025;25(1), 1259. https://doi.org/10.1186/s12889-025-22036-4

**Social Support Rating Scale (SSRS)**

**Instructions: Hello! The following questions are used to reflect the support you receive in society. Please write according to the specific requirements of each question and your actual situation. Thank you for your cooperation!**

**1.How many close friends can you get support and help from? (Choose only 1 item)**

(1) None at all (2) 1-2 (3) 3-5 (4) 6 or more

**2.In the past year, have you: (Choose only one)**

(1) Been away from family and lived alone (2) Frequently changed your residence, mostly living with strangers

(3) Lived with classmates, colleagues, or friends (4) Lived with family.

**3.You and your neighbors: (Choose only one)**

(1) Never care for each other, just nodding acquaintances (2) May care a little when in trouble

(3) Some neighbors care about you (4) Most neighbors care about you

**4.You and your relatives: (Choose only one)**

(1) Never care for each other, just nodding acquaintances (2) May care a little when in trouble

(3) Some colleagues care about you (4) Most colleagues care about you

**5.Support and care received from family members (Check the appropriate box)**

|  | None | Very little | Average | Full support |
| --- | --- | --- | --- | --- |
| A. Spouse (lover) |  |  |  |  |
| B. Parents |  |  |  |  |
| C. Children |  |  |  |  |
| D. Siblings |  |  |  |  |
| E. Other members (such as sisters-in-law, etc.) |  |  |  |  |

**6.In the past, when you were in urgent and difficult situations, the sources of financial support and help in solving practical problems you have received are:**

(1) No source at all.

(2) There are sources, which are the following: (Multiple options can be selected)

A. Spouse; B. Other family members; C. Friends D. Relatives;

E. Colleagues; F. Workplace; G.Party and mass organizations, such as trade unions, etc., official or semi-official organizations, H. Religious, social group organizations, etc., non-official organizations: H.Others (please list)

**7.In the past, when you were in urgent and difficult situations, the sources of comfort and care you have received are:**

(1) No source at all.

(2) There are sources, which are the following: (Multiple options can be selected)

A. Spouse; B. Other family members; C. Friends D. Relatives; E. Colleagues;

F. Workplace;

I.Party and mass organizations, such as trade unions, etc., official or semi-official organizations, H. Religious, social group organizations, etc., non-official organizations:

J.Others (please list)

**8.Your way of confiding when you encounter troubles: (Choose only one)**

(1) Never confide in anyone: (2) Only confide in 1-2 very close people

(3) If friends take the initiative to ask, you will speak out. (4) Actively confide your troubles to gain support and understanding

**9.Your way of seeking help when you encounter troubles: (Choose only one)**

(1) Rely only on yourself and do not accept help from others (2) Seldom ask for help from others

(3) Sometimes ask for help from others (4) Often ask for help from family, friends, and organizations when in trouble

**10.Regarding group (such as party organizations, religious organizations, trade unions, townships, village committees, etc.) organized activities, you: (Choose only one)**

(1) Never participate (2) Occasionally participate (3) Often participate (4) Actively participate and actively engage

**Detailed Description of the Social Support Rating Scale (SSRS)**

**Introduction to the Scale**

The Social Support Rating Scale (SSRS) is a widely used instrument for evaluating the level of social support among individuals. It was developed to measure the perceived quality and quantity of social support from family, friends, and other significant relationships.

Structure of the Scale

**The SRS scale consists of items that assess three main dimensions of social support:**

**Subjective Support** (Subjective): Item 1 + Item 2 + Item 3 + Item 4 + Item 5

**Objective Support** (Objective): Item 6 + Item 7 + Item 8

**Utilization of Support** (Utilization): Item 9 + Item 10 + Item 11

Each dimension is designed to capture different aspects of social support, providing a comprehensive assessment of an individual’s social environment.

**Scoring Method**

**Scoring Criteria:** Each item is scored on a 5-point scale, with responses ranging from "very poor" to "very good" or "never" to "always".

**Special Cases:**

Item 6 and Item 7 are scored based on the frequency of support received, with higher scores indicating more frequent support.

Item 8 assesses the satisfaction with the support received, with higher scores reflecting greater satisfaction.

Item 9 and Item 11 evaluate the perceived helpfulness and availability of support, respectively.

**Total Score Calculation:**

**Subjective Support Score (Subjective)**: Sum of all subjective support items.

**Objective Support Score (Objective):** Sum of all objective support items.

**Utilization of Support Score (Utilization):** Sum of all utilization of support items.

**Total Social Support Score**: Total score = Subjective + Objective + Utilization.

**Total Score Classification**

According to the total score calculation formula of the SRS scale, the final score ranges from 12 to 66 points:

12-23 points: Low social support, which may indicate a lack of adequate social resources.

23-44 points: Moderate social support, suggesting a reasonable level of social interaction and support.

45-66 points: High social support, indicating strong social networks and high satisfaction with social relationships.

**Reliability and Validity of the Scale**

**Reliability:** The SSRS scale has high internal consistency, with a Cronbach's α coefficient typically ranging from 0.70 to 0.90.

**Validity:** The scale demonstrates good validity across different cultural backgrounds and age groups, making it a reliable tool for assessing social support in various populations.

**References**

1. Xiao S. Y. Theoretical basis and research application of Social Support Rating Scale (SSRS). J. Clin. Psychiatry 1994;4: 98–100.
2. Zheng Z, Han W, Li Y. The mediating role of coping style in the relationship between depression and disordered eating among Chinese female undergraduates. Front. Psychol. 2019;10:3011.

**Geriatric Depression Scale (GDS-15)**

**Instructions: Hello! The following questions are used to reflect your feelings about life. Please check the box that fits your actual situation according to the specific requirements of each question, thank you for your cooperation!**

| **Number** | **Choose the answer that fits how you have felt over the past week** | **Yes** | **No** |
| --- | --- | --- | --- |
| 1 | Are you basically satisfied with your life? | 0 | 1 |
| 2 | Have you given up many of your usual activities and interests? | 1 | 0 |
| 3 | Do you feel that your life is not full enough? | 1 | 0 |
| 4 | Do you often feel annoyed? | 1 | 0 |
| 5 | Do you often feel mentally good? | 1 | 0 |
| 6 | Are you worried that something bad will happen to you? | 1 | 0 |
| 7 | Do you often feel happy? | 1 | 0 |
| 8 | Do you often feel lonely or helpless? | 0 | 1 |
| 9 | Would you rather stay at home than do things you are not familiar with? | 1 | 0 |
| 10 | Do you feel your memory is worse than other elderly people? | 0 | 1 |
| 11 | Do you think it's great to have lived this long? | 1 | 0 |
| 12 | Do you feel useless? | 0 | 1 |
| 13 | Do you feel energetic? | 0 | 1 |
| 14 | Do you feel that your situation is hopeless? | 1 | 0 |
| 15 | Do you feel that most people are richer than you? | 1 | 0 |

**GDS-15 Depression Scale:** This scale consists of 15 items with a 4-point rating. The higher the score, the more severe the depressive symptoms. A score of 0-5 indicates no depressive symptoms; 6-10 indicates mild depressive symptoms; 11-15 indicates severe depressive symptoms.

**Detailed Description of the Geriatric Depression Scale (GDS-15)**

**Introduction to the Scale**

The Geriatric Depression Scale (GDS-15) is a widely recognized tool designed to assess depressive symptoms in older adults It was developed to be sensitive to the unique characteristics of depression in the elderly, such as cognitive impairments and somatic complaints.

**Structure of the Scale**

The GDS-15 consists of 15 items, Each item is designed to capture specific aspects of depressive symptoms commonly experienced by older adults.

**Scoring Method**

**Scoring Criteria:** Each item is scored on a 0-3 scale, with higher scores indicating more severe symptoms.

**Total Score Calculation:**

Total Score: Sum of all 15 items.

The total score ranges from 0 to 45 points.

**Total Score Classification**

According to the total score calculation formula of the GDS-15 scale, the final score ranges from 0 to 45 points:

0-5 points: Minimal or no depression.

6-10 points: Mild depression.

11-15 points: Moderate to severe depression.

**Reliability and Validity of the Scale**

**Reliability:** The GDS-15 has high internal consistency, with a Cronbach's α coefficient typically ranging from 0.80 to 0.90[1].

**Validity:** The scale demonstrates good validity across different cultural backgrounds and age groups.

**References**

1. Yesavage JA, Brink TL, Rose TL, Lum O, Huang V, Adey M, et al. Development and validation of a geriatric depression screening scale: a preliminary report. J Psychiatr Res. 1982;17(1):37–49^[4^].
2. Tang WK, Wong E, Chiu HF, Lum CM, Ungvari GS. The Geriatric Depression Scale should be shortened: results of Rasch analysis. Int J Geriatr Psychiatry. 2005 Aug;20(8):783–789^[9^].
